# A study of mapping usual care and unmet need for vocational rehabilitation and psychological support following major trauma in five health districts in the UK

**DOI:** 10.1177/0269215520971777

**Published:** 2020-11-23

**Authors:** Jade Kettlewell, Stephen Timmons, Kay Bridger, Denise Kendrick, Blerina Kellezi, Jain Holmes, Priya Patel, Kate Radford

**Affiliations:** 1 Centre for Health Innovation, Leadership and Learning, University of Nottingham Business School, Nottingham, UK; 2 Division of Primary Care, University of Nottingham School of Medicine, Nottingham, UK; 3 Nottingham Trent University, School of Social Sciences, Division of Psychology, Nottingham, UK; 4 Division of Rehabilitation, Ageing and Wellbeing, University of Nottingham School of Medicine, Nottingham, UK; 5 Centre for Neuroscience, Surgery and Trauma, Queen Mary University of London, London, UK

**Keywords:** Traumatic injury, vocational rehabilitation, psychological support, major trauma centre, Unmet needs

## Abstract

**Objective:**

To identify where and how trauma survivors’ rehabilitation needs are met after trauma, to map rehabilitation across five UK major trauma networks, and to compare with recommended pathways.

**Design:**

Qualitative study (interviews, focus groups, workshops) using soft-systems methodology to map usual care across trauma networks and explore service gaps. Publicly available documents were consulted. CATWOE (Customers, Actors, Transformation, Worldview, Owners, Environment) was used as an analytic framework to explore the relationship between stakeholders in the pathway.

**Setting:**

Five major trauma networks across the UK.

**Subjects:**

106 key rehabilitation stakeholders (service providers, trauma survivors) were recruited to interviews (*n* = 46), focus groups (*n* = 4 groups, 17 participants) and workshops (*n* = 5 workshops, 43 participants).

**Interventions:**

None.

**Results:**

Mapping of rehabilitation pathways identified several issues: (1) lack of vocational/psychological support particularly for musculoskeletal injuries; (2) inconsistent service provision in areas located further from major trauma centres; (3) lack of communication between acute and community care; (4) long waiting lists (up to 12 months) for community rehabilitation; (5) most well-established pathways were neurologically focused.

**Conclusions:**

The trauma rehabilitation pathway is complex and varies across the UK with few, if any patients following the recommended pathway. Services have developed piecemeal to address specific issues, but rarely meet the needs of individuals with multiple impairments post-trauma, with a lack of vocational rehabilitation and psychological support for this population.

## Introduction

Traumatic injuries in working age adults are a global public health problem. Traumatic injury or ‘major trauma’ describes serious and often multiple injuries where there is a strong possibility of death or disability^1^ (e.g. traumatic brain injuries, complex fractures). Survivors of such injuries may experience physical, social and psychological problems, such as pain, fatigue, depression and anxiety, interpersonal difficulties, or hidden disabilities, such as cognitive problems. A significant number of people experiencing trauma have residual problems affecting their ability to return to, and remain in, work.^2,3^ Therefore, it is important that rehabilitation to support these individuals addresses all issues long-term.

Systematic reviews suggest that vocational rehabilitation improves return-to-work for some conditions, such as brain and spinal cord injury,^4–6^ back pain^7^ and mental health problems.^8^ However, moderate to severe trauma can affect single or several body regions, which frequently leads to long-term psychological problems.^9^ Whilst there is evidence that effective vocational rehabilitation for some types of injuries such as brain and spinal cord injury addresses both physical and psychological problems,^5,7,10^ evidence of this for orthopaedic injury is lacking.^11,12^ Previous studies have not evaluated vocational rehabilitation in complex multi-organisational settings, such as UK National Health Service (NHS) major trauma centres, which receive traumatic injury patients from large geographical areas and repatriate patients to a wide range of local services.

Rehabilitation service organisation is in most countries, complex with multiple organisations involved. This means that rehabilitation pathways are not always consistent and are challenging to standardise and evaluate, even in the UK, which has a universal integrated healthcare system. The British Society of Rehabilitation Medicine core standards for specialist rehabilitation following major trauma state that individuals should have access to specialist vocational rehabilitation services in the UK.^13,14^ Although published standards highlight the need for support to return-to-work following trauma, there is limited evidence describing the consistency and quality of service provision across the UK. Studies mapping vocational rehabilitation for specific conditions, such as stroke^15^ and long-term neurological conditions^16,17^ highlight the disparity between service provision in different regions of the UK.

In this study, we aimed to: (1) understand where and how trauma survivors’ rehabilitation needs are currently met in the UK trauma pathway in terms of vocational and psychological support and; (2) map current UK NHS rehabilitation (usual care) across five trauma networks. A trauma network is the collaboration between providers commissioned to deliver trauma care services in a geographical area.

## Methods

This study feeds into a larger programme of work funded by the National Institute for Health Research (NIHR, Ref: RP-PG-0617-20001). Findings will inform the development and implementation of a return-to-work intervention (www.ROWTATE.org.uk). Ethical approval was obtained from the University of Nottingham Faculty of Medicine and Health Sciences Research Ethics Committee (Ref: FMHS 150-1811) and Leicester South NHS Research Ethics Committee (Ref 19/EM/0114). Recruitment lasted 12 months, starting in February 2019.

Participants were recruited using purposive sampling via the University of Nottingham research team, and via Principal Investigators at five UK major trauma centres. Participants were also recruited through existing contacts and known providers of rehabilitation services. Written informed consent was obtained from all participants taking part in audio recorded interviews and focus groups. For all other research activities (informal interviews, workshops), participants were given the time and opportunity to opt out, otherwise consent was assumed.

Researchers (authors JK, KB) conducted semi-structured interviews (n = 38) and focus groups (*n* = 4 focus groups, total of 17 participants) with stakeholders to obtain qualitative data about the rehabilitation pathways. Workshops (*n* = 5 workshops, total of 43 participants) with service providers and trauma survivors (rehabilitation physicians, occupational therapists, physiotherapists, speech and language therapists, psychologists, nurses, trauma practitioners and previous trauma patients) were also conducted by researchers (JK, ST) at five UK major trauma centres to understand more about their trauma pathway. For further information about the pathways, appropriate service providers (e.g. trauma practitioners, case managers, trauma rehabilitation coordinator) were consulted through informal interviews (*n* = 8) about current referral processes and usual care within these centres. The data were used to map current rehabilitation pathways and create a rich description of usual care across the five major trauma centres. Publicly available documents/online resources (*n* = 10) and relevant NHS Trust website were also consulted. These included the National Clinical Audit of Specialist Rehabilitation for Patients with Complex Need following Major Injury reports, British Society of Rehabilitation Medicine recommendations for best practice and the Trauma Audit & Research Network website. Summary of resources are shown in Supplemental Appendix 1.

We followed Sinclair’s^15^ approach to using soft-systems methodology to guide the data collection and analysis of this study. We used an analytic soft-systems methodology framework known as CATWOE (Customers, Actors, Transformation, Worldview, Owners, Environment), to guide the interview questions and inform data coding and analysis (Table 1).^18^ This framework enabled us to generate an operational definition of usual care in the major trauma centres allowing understanding of where needs are met and to map existing services. It also allowed us to understand the relationship between those delivering services (actors), such as vocational rehabilitation therapists and those receiving usual care (customers), such as trauma survivors.

**Table 1.** Summary of CATWOE definitions.

CATWOE	Definition	Relevance to research aim
Customers	Patients receiving usual care, or the beneficiaries of the system.	Trauma survivors, family members or other stakeholders (e.g. employers) benefitting from usual care rehabilitation and vocational support.
Actors	People delivering rehabilitation and providing care.	Service providers (therapists, psychologists, occupational health, GPs, rehabilitation consultants, occupational health, physicians) providing the vocational rehabilitation or supporting a person in their return to work.
Transformations	Changes occurring as a result of usual care and additional services.	Communication between therapist and employer, or patient and employer to initiate the return to work process. Actions taken by key stakeholders.
World View	Context in which the transformation is meaningful, evaluation and knowledge of services.	Views, beliefs and opinions of those involved in the return to work process such as the patient, therapist and employer. The influence the key stakeholders have on the process.
Owners	Who the service is answerable to or funded by, who could stop changes from occurring.	Those that could affect the success of a return to work, in most cases those commissioning services.
Environmental context	Contextual, political and physical factors that may influence services.	The context in which the return to work process needs to occur. Potential environmental and contextual barriers (e.g. geography, resources) in respect of service provision, workplace or support.

Most interviews and focus groups were audio recorded where possible and transcribed, otherwise notes were taken. Workshops were recorded using contemporaneous notes by a researcher. All data were analysed thematically following Braun and Clarke’s^19^ approach, informed by the a priori constructs of CATWOE. The transcripts were independently analysed by authors (JK and KB) and main themes identified were discussed with other authors (KR, ST) for agreement. Data were also used to inform the mapping of service pathways.

**Figure 1.**
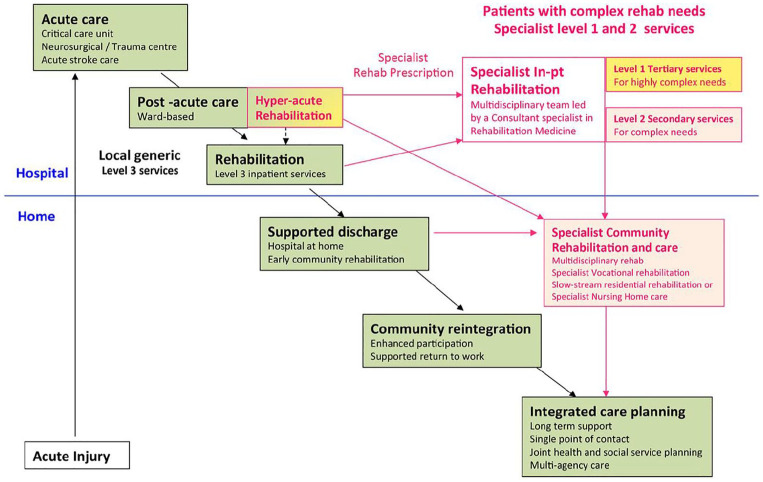
The ‘ideal’ rehabilitation pathway following major traumatic injury taken from the BSRM core standards.^14^ Patients flow through the system from acute care to community care, sometimes requiring more specialist care at a level 1 or 2 inpatient unit.

Rehabilitation pathways were visually mapped across five major trauma networks, which were informed by consultation with stakeholders (e.g. rehabilitation consultants, therapists, clinical psychologists, NHS managers, solicitors), as previously mentioned. In order to highlight the complexity of the system, ensure consistency of reporting and enable comparison across the different trauma networks, the pathways were mapped against the British Society of Rehabilitation Medicine ‘Core Standards for Specialist Rehabilitation following Major Trauma’. This ‘ideal’ pathway post-trauma is shown in Figure 1.

## Results

We recruited a variety of key stakeholders (*n* = 106) including trauma survivors, carers, NHS service providers (e.g. case managers, general practitioners and other trauma rehabilitation specialists) private service providers and solicitors. Some participants participated in interviews and focus groups. A summary of the characteristics of participants is shown in Table 2. Service providers worked across different NHS healthcare settings, including acute, community and primary care, private rehabilitation providers, third sector services and the insurance industry.

**Table 2.** Characteristics of participants.

Participant type	Injury/profession	Number	% total (*n* = 106)^†^
Trauma survivor	Amputation	1	1
	Brain Injury	6	6
	Orthopaedic	10	9
	Poly-trauma (including brain injury)	2	2
	Spinal injury	2	2
Carer	Partner with orthopaedic injury	1	1
	Partner with traumatic injury	1	1
Healthcare provider	Case manager	3	3
	Clinical psychologist	10	9
	Emergency doctor/consultant	4	4
	General Practitioner	4	4
	Occupational physician	1	1
	Occupational psychologist	1	1
	Occupational therapist	26	25
	Physiotherapist	5	5
	Psychiatrist	1	1
	Rehabilitation doctor/consultant	12	11
	Speech and language therapist	1	1
	Trauma rehabilitation coordinator	1	1
	Trauma practitioner	4	4
	Trauma psychologist/psychotherapist	2	2
Other stakeholder	Clinical researcher	1	1
	Disability employment advisor	3	3
	Solicitor	2	2
	Trauma charity volunteer/coordinators	2	2

† percentages rounded to nearest whole number, hence does not sum to 100%.

Data obtained through qualitative methods and extensive pathway mapping highlighted the complexity of the trauma pathways across England. Some of the common issues identified across the major trauma networks were: (1) inconsistent transition from acute care to community services due to a lack of communication between different services and healthcare providers when a patient is discharged from hospital; (2) geographical barriers (e.g. postcode lottery); (3) a lack of expertise in areas located further from the major trauma centre; and (4) a clear gap in vocational and psychological support for trauma survivors, particularly those with musculoskeletal injuries.

**Figure 2.**
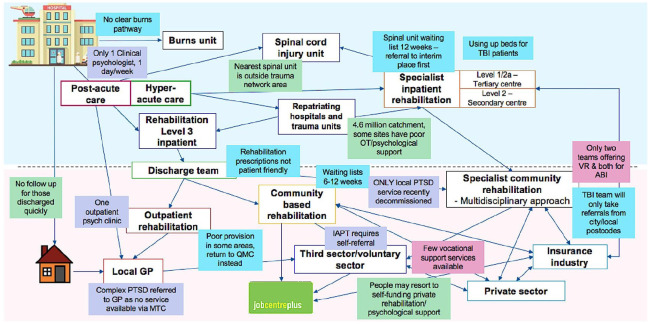
The ‘reality’ of the trauma pathway; example of rehabilitation pathway, highlighting the current issues. ABI: acquired brain injury; GP: general practitioner; IAPT: improving access to psychological therapies; MTC: major trauma centre; TBI: traumatic brain injury; PTSD: post-traumatic stress disorder; VR: vocational rehabilitation.

The mapped pathways for each major trauma network are shown in Supplemental Appendices 2 to 6. It is clear that the ‘ideal’ flow of patients through the rehabilitation system as depicted by the British Society of Rehabilitation Medicine guidelines (Figure 1), is not the case in the five networks illustrated. It is in fact, much more complex, with some patients receiving no support or being referred to an inappropriate facility (e.g. spinal cord injury patient being repatriated to a brain injury unit whilst waiting for an appropriate bed space). However, it is important to note that some well-established pathways that sufficiently support trauma survivors do exist, but the majority of these pathways are for patients with neurological conditions. A summary of one pathway is shown in Figure 2, highlighting the issues at each point along the pathway. There is a clear difference to the ‘ideal’ trauma pathway.

The use of the analytical framework (CATWOE) facilitated the understanding of the implementation context for trauma rehabilitation services and the issues within the current pathway.

**Customers**: Customers are anyone that could benefit from vocational rehabilitation/psychological support, which could be the trauma survivor, their family, or their employer. Individuals often have unrealistic expectations of their recovery post-trauma, which causes stress to the patient and in some cases, the employer:

*‘I’ve got many, many patients who they ignore our [rehabilitation experts] advice and go back to work earlier, and they go a step backwards’ (Rehabilitation consultant)*

*‘I think I should have taken on some reduced duties or something first. That was my choice. They [employer] offered that and I said I’d be fine and then it turned out pretty bad for me.’ (Trauma survivor, musculoskeletal injury)*

There is also a general lack of knowledge outside of the healthcare system about the impact of trauma, which makes the return-to-work process even more challenging:

*‘There is that big question around disclosure, head injuries, trauma, for ‘Joe Bloggs’ employers it’s quite a hard thing to get their heads round, well hang on a minute how is this [injury] going to impact on you [patient]?. . . sometimes a lot of employers, they don’t know, they haven’t got a clue, they have never had to deal with it, they don’t know where to go, they don’t know where to ask [for support].’ (Disability employment advisor)*

There is a clear gap in vocational support for individuals with certain types of injuries, especially traumatic amputations, and musculoskeletal injuries:

*‘With orthopaedic, complex orthopaedic stuff, which is going to be the vast majority I think of what you’re seeing here, occupational therapy provision is difficult I think, it’d be fair to say. . .and there isn’t an occupational therapy vocational rehab service for these people.’ (Rehabilitation consultant)*

*‘Unless you have quite a bad head injury, they [commissioners] just don’t see musculoskeletal injuries as a problem or amputee people don’t think they need help to go back to work because they just crack on, but actually there may be things [that are required], like worksite assessment.’ (Occupational therapist)*

A number of service providers were unaware of specific support for amputees and people with burns, by comparison with the well-known charities for brain and spinal injuries. The majority of vocational rehabilitation teams across the five major trauma pathways only offer return-to-work support to individuals with acquired or traumatic brain injury (See Supplemental Appendices 2–5). Thus, service providers perceive that more patients with brain injury tend to get back to work than those with spinal injury, even though the latter typically have no cognitive impairment:

*‘Because I think there’s actually a lower percentage of spinal clients that get back to work than there is brain injury. . .where you have targeted neuro rehab but focused on vocational, you could be looking to get sort of 30, 33% back to work whereas I think spinal is something like 19 or 20%, which is interesting when they’re cognitively intact in the main. So, you would think that the barriers would be more physical in nature but they don’t get returned’ (Case manager)*

Stakeholders suggested that the pathway is currently skewed in favour of those with acquired brain injuries, perhaps because they often require more intense, long-term rehabilitation. Stakeholders also expressed concern over the lack of support for individuals that are discharged quickly from hospital, with seemingly less severe injuries. These individuals move through the trauma pathway so quickly that they do not get picked up by community-based services.

Some individuals do not want their employers to know about psychological issues or outcomes of injury that are not obvious, such as fatigue, incontinence, cognitive problems and anxiety. This makes it particularly challenging for therapists when supporting someone in their return-to-work, as such issues, if unaddressed, may act as barriers to a sustainable return-to-work:

*‘. . .most people with considerable, significant physical injuries focus on recovering the physical component of their injuries, not recognising the psychological aspects which themselves are neglected by the patients or their carers, and/or their staff, but are only picked up once the patient tries to reintegrate into society’ (Clinical psychologist)*

**Actors**: The individuals delivering care play an important part in the success of rehabilitation and an individual’s return-to-work. However, referrals are impacted by lack of knowledge about which services currently exist and service providers’ understanding of the whole rehabilitation pathway:

*‘One of the discussions that we’ve just had is, we, as a service, are very unaware of exactly what’s available in the county. . .We met recently with representatives from some of these teams, the brain injury team, etc, and I think the issue at the moment is that actually it’s very much a patchwork of availability. (Clinical psychologist)*

Although some stakeholders had better knowledge of service provision in their local area, this tended to be condition-specific and relevant to their position in the pathway (e.g. an acute hospital-based occupational therapist will be aware of equipment provision for discharge planning, but may not know about vocational support). There is a general lack of knowledge about vocational rehabilitation and psychological services across the different trauma networks.

Employers face similar challenges and may not always recognise psychological or hidden issues, making it difficult for them to understand how to support an employee in their return-to-work. They often require the help of a therapist to make workplace adjustments:

*‘Managing those very difficult conversations because employers will say, can this person do the job or not, and they’re asking you as a clinician to make that decision. And I think one of the useful things I’ve learnt from our work psychologist was, you don’t say, yes or not, because you’re not the employer. . .sometimes I have said, you’re the employer, these are the things that would support this person to be able to do this job.’ (Vocational rehabilitation occupational therapist)*

Another challenge raised by stakeholders was the difficulty in finding an appropriate role for someone after traumatic injury and the importance of the NHS service provider (e.g. occupational therapist) in supporting the employer with this process:

*‘I think part of the difficulty is trying to work out how you can change work and getting the employers to think differently about why they should support somebody going back to work, particularly I think in high demand and highly technical jobs or very physical jobs. You often find that the employee needs an alternative job that they don’t have the skills to do anything else and that’s very difficult.’ (Occupational physician)*

Some stakeholders stated that patients are not routinely asked about mood. One therapist highlighted the challenge of supporting someone with psychological needs who does not want to engage with therapy and the issue of not always being aware of such needs:

*‘. . .depending on the client, we word it different ways but it’s essentially how’s your mood? But as you know, people can’t engage in therapy if they – if we’re not addressing their mental health because they might be so depressed that they’re not opening their letters, so they don’t know when their appointment is. They can’t organise themselves to get out of bed to arrange transport to get in here. It’s [addressing mental health needs] like the nuts and bolts of what we do; we can’t do what we do without being aware of that.’ (Speech and language therapist)*

A common reason for not asking patients about their mental health or return-to-work plans was a lack of confidence in dealing with these issues (i.e. not wanting to probe these important issues in case they cannot support the patient), or limited referral options should individuals require more specific support even when feeling confident about asking:

*‘I think it depends if you’ve got an interest in it [vocational rehabilitation] as well. . .If you feel confident. And what we’re [senior therapists] finding. . .is a lot of OTs aren’t feeling confident about asking that question [about returning to work], they find it quite scary.’ (Occupational therapist)*

Lack of service provision means that therapists are avoiding addressing issues that they cannot deal with:

*‘There’s just not really the [vocational or psychological] services out there to then signpost people on to. So, you almost feel like you’re opening a Pandora’s box where you can’t actually then put those pieces back in.’ (Trauma ward occupational therapist)*

**Transformations:** Stakeholders suggested that there is a lack of effective communication between service providers, patients, and employers when a patient moves from the acute setting to the community. Trauma survivors felt there was poor continuity of care and consistency in support after they left the major trauma centre. Multiple service providers described the system as a lottery. This means that some individuals fall through gaps in the system, receiving little or no support:

*‘I mean it’s part of the problem, it’s not coordinated, it’s a bit of a lottery as to what the pathway was when you came through the service, what your injuries were, which directorate you came through, what your postcode is, it’s a lottery to what you can access. Some get really good stuff; seen some really good UKROC [UK specialist Rehabilitation Outcomes Collaborative] rehab. . .but then people have to wait four months to see an OT. It’s good when you get it.’ (Rehabilitation consultant)*

In addition, therapists stated how hard it was to keep track of whether onward referrals for trauma survivors were successful and concerns over whether their patients were receiving appropriate rehabilitation following discharge:

*‘When I was a ward therapist, you’d refer them [patients] and you didn’t know if they were then accepted, or if that referral had got missed. So, you’d just refer them in good faith, and you don’t know then what happened or if you’re referring to somewhere else’ (Community occupational therapist)*

*‘There’s just nobody to take on that role for even making sure referrals have actually gone through to these places [community teams]. And again, the numbers change from week to week and the services change week to week. There’s no central point of access for any of it [referral success]. (Hospital occupational therapist)*

The issue of limited resources causing long waiting lists was raised by the majority of stakeholders as one of the barriers to patients receiving vocational rehabilitation. Waiting lists for community rehabilitation are long across the five trauma networks, and in some areas, up to 12 months. Along with extended waiting lists, there are few services providing vocational support within each trauma network, meaning that these community teams are overloaded with referrals and do not have the resources to deal with the many individuals requiring vocational and psychological rehabilitation, and are not receiving the timely support they require:

*‘. . .and again it’s about the time scale and it’s about the availability of resources. A health care district might only have one neuro psychologist to deal with everything so it’s how thin can they spread themselves really?’ (Case manager)*

**World view**: The public and professional view of vocational rehabilitation has an influence on the delivery and funding of such services, which ultimately has a large impact on whether individuals are supported in their return-to-work following trauma.

Various service providers discussed the view that delivering vocational rehabilitation in the acute setting is often seen as unimportant by acute physicians and therapists. Attitudes among some healthcare providers suggests that starting vocational rehabilitation in acute setting is ‘too early’ and is not discussed, even though some are aware of the evidence supporting the benefit of early intervention^20–25^:

*‘People don’t know their rights about return to work or remaining in work. It’s a big problem. People don’t even mention work in an acute hospital. It just doesn’t even get discussed.’ (Occupational therapist)*

*‘I think that focus is not in the mind of acute trauma team. I think in order to get it – keep that focus this [do you want to get back to work] needs to be said from day one and day two and we keep that over time, this chap is a driver, this chap is a butcher or something. We don’t do that in the NHS unfortunately.’ (Rehabilitation consultant)*

In the initial stages post-injury, the focus is primarily on treating the medical issues and ensuring the patient can be discharged from the major trauma centre as safely and as soon as possible. However, this means that often the biopsychosocial factors that are important to an individual’s recovery are sometimes overlooked, especially their need to return-to-work.

*‘Return to work is a luxury not a necessity’ (Occupational therapist)*

A ‘world view’ where supporting return-to-work is seen as an extravagance and not routinely provided, shows poor understanding of why vocational rehabilitation can be appropriate for those who want to work, but may struggle in their pre-injury role. Some service providers suggested that employers do not know how or are unwilling to support someone whose abilities have radically altered and can only, for example, work a few hours per week. Being able to gradually reintegrate into work and accomplish a meaningful amount of work whilst there is even more of a challenge when an employer is not willing to support their employee:

*‘If people can’t get back to work within four to six weeks, they [employers] will not start a phased return. . . people pull their hair out when they get hospitals or GPs say, this person can only work two hours a week. They’re like, there’s no point. There’s no job that can be done for two hours a week.’ (Occupational physician)*

**Owners**: The provision of vocational rehabilitation, or any rehabilitation services are strongly influenced by government policies and those commissioning such services. This could include policy makers, service managers and commissioners (someone involved in the planning and purchase of NHS and publicly funded social care services) but may also include managers in an employing organisation and Occupational Health or Human Resources departments.

One of the issues with ensuring an individual receives appropriate support post-trauma is keeping track of services which are constantly changing in response to funding alterations and service developments. Even when good services exist, service providers frequently expressed their frustration over instances of the decommissioning of vocational rehabilitation services, which are already limited. One occupational therapist explained that decommissioning often happens if the need of such a service is not recognised from a commissioning perspective:

*‘That’s one of the problems, constant change. And we used to have a super vocational rehab service in our community team and they got rid of it because they [commissioners] didn’t think people needed to return to work’ (Occupational therapist)*

This view was corroborated by others:

*‘There’s a lot of need, and not a lot of provision. . . [rehabilitation] is the first thing to get axed when budget cuts come in.’ (Rehabilitation consultant)*

The perceived value for money of the service can determine which services are funded (i.e. services appear to apply to a small percentage of a population locally or services that are not strongly evidence-based). This can impact especially those with more severe trauma who need longer term support, including with psychological issues:

*‘So I think that’s another thing I notice. . . is that hospitals are forever trying to get rid of follow-up clinics. . . you’re not going to spot recurrence [of psychological issues] when you see someone in an outpatient clinic so let’s just get rid of them. It’s just so expensive in doctor time. . .But obviously psychologically, it’s really important time for continuity of relationships.’ (Clinical psychologist)*

There was a perception that those responsible for making service delivery plans do not always understand the medical diagnoses leading to poor service design and funding decisions, ineffective rehabilitation, and non-individualised care. One stakeholder highlighted the challenges for trauma survivors where needs are not met because services are designed to match resources. Some people fall outside the service referral criteria, but have rehabilitation needs that require support, some are only able to receive rehabilitation for a finite period but is required for a longer duration:

*‘As resources have become more limited within the NHS, you find that things have become much more streamlined so people are only offered so many weeks of service intervention or in order to qualify for some brain injury services you have to have more than two different problems. So, if you need cognitive rehab and you need speech and language therapy you’ll get a service. But if you only need cognitive rehab it’s more difficult.’ (Case manager)*

**Environmental context**: The major trauma centres across the UK are located in major cities and tend to be surrounded by multiple community-based teams, with easier access to support. However, further away from the trauma centre, service provision becomes patchy and there is greater inconsistency in the availability of psychological and vocational support. Each major trauma centre is considered the hub of its trauma network and repatriating hospitals are seen as the spokes. However, between the hub and the spokes can be up to two hours travelling distance, making it challenging for patients to access the major trauma outpatient services. There is also geographical disparity across the country in terms of what is available for individuals post-trauma. Often those located in more rural areas receive the least support or must travel long distances to receive support. One trauma survivor highlighted their experience of leaving the acute setting:

*‘One of the areas I struggled with is when you’re in the major trauma centre, you very quickly become aware that you are getting the best treatment that is available, but of course as soon as you become well enough, they want to move you to either your local hospital or something like that, which is what I did. I actually did feel – I actually feel that the physio and everything just went down a notch, not in a bad way but in a noticeable way.’ (Trauma survivor, polytrauma)*

Inconsistent service provision across the country and constantly changing services means that the system is challenging for service providers to navigate. Stakeholders stated that services either do not exist, had been recently decommissioned, or the team was spread so thinly across the region that they were unable to see new patients:

*‘So, in terms of geographical area, [county name] is split into North and South [county name] for a lot of services, and some services cover both parts, some just the north, some just the south. The service that they do offer is often very different in terms of assessment, treatment, how long people can be treated for, things like that. . .So it’s a real patchwork. . .there’s a lot of uncertainty.’ (IAPT Clinical psychologist)*

As service provision is limited in some areas, stakeholders frequently stated that patients are often offered outpatient appointments at their major trauma centre, so that they receive timely support, even if it is a significant distance to travel. Often no local services are available, so patients opt to return to the major trauma centre, which may be difficult for individuals:

*‘The community services are very fragmented, and again a lot of what I see is sort of more specialist neuro rehab stuff, but similarly, you know, we end up bringing people back to physio in [city name] sometimes, if they’re struggling to access, even just for orthopaedic physio, and particularly the less experienced, if they’ve got really complex, like pelvic injury, all those kind of things.’ (Rehabilitation consultant)*

## Discussion

Our findings support the hypothesis that the rehabilitation pathways followed by patients after trauma are extremely complex, with few, if any patients following the proposed ideal pathway put forward by the British Society of Rehabilitation Medicine.^14^ Although individual services aim to deliver effective rehabilitation, the lack of communication between acute and community services has an impact on the continuity of care being provided.

There are several well-established pathways, particularly for people with neurological injuries, however there are clear gaps in service provision for those with musculoskeletal injuries and amputations. There is a consistent lack of vocational rehabilitation and psychological services across the major trauma networks, and in areas where they do exist, the waiting lists are too long to provide timely support. Although some of the issues within the pathway are common knowledge among trauma stakeholders, there is limited evidence explicitly highlighting the disparity in service provision across different regions of the UK.

Our study supports prior UK studies with traumatic injury patients, which highlighted the lack of psychological discussion, support and signposting for trauma patients,^26^ and gaps identified when transitioning from hospital to community.^27^ Previous research also corroborates our findings in that clinical decisions post-trauma are limited by insufficient resources, gaps in communication, conflicting organisational priorities and unrealistic patient expectations,^28^ and highlights the value of effective multi-disciplinary input and co-ordinated care.^29^ However, this study systematically explored these issues and the extent of current provision gaps in five UK major trauma centres, providing a more detailed map of current service provision that no prior research has achieved to date.

The present work also highlights the geographical nature of the gaps and how these impact on service access. This study contributes to the evidence gap in understanding UK service provision across the pathways and identifies geographical areas and services that require more funding. Until awareness is raised about geographical areas across the UK with limited service provision and service gaps, it is unlikely that change will occur and thus, the system will not improve. In addition, this work may contribute to the understanding of international rehabilitation services with similar integrated systems to the UK NHS, highlighting areas that may require more funding or support, or potentially inform pathway development in other complex healthcare systems.^30^

Crossing boundaries (i.e. across healthcare, social care, industry, employment sector) and multidisciplinary delivery of rehabilitation is important,^31^ particularly when providing vocational and psychological support.^7,10,32,33^ However, there still appears to be a lack of communication and continuity of care when patients are discharged from an acute setting. Although a known issue among clinicians and reported across the literature,^26,27,29,34^ our findings suggest that problems still exist. The introduction of the Rehabilitation Prescription in 2013 aimed to improve communication along the pathway and ensure that all information concerning injury management (including long-term goals) is transferred across all relevant services/sectors. The 2019 NSCASRI report stated that even though the Rehabilitation Prescription was completed for 89% patients, the mandated data collection was limited (physical, cognitive/mood, psychosocial needs) providing little useful information about rehabilitation needs.^35^ An updated version of the Rehabilitation Prescription was released in 2019 and hopes to address this gap in continuity of care following discharge, by requiring a summary of rehabilitation needs on leaving the acute setting.^36^

Geographical barriers are an issue within the current rehabilitation pathway, and service provision tends to become more limited the further a patient is located from a major trauma centre. Some major trauma centres have up to two-hour repatriation distances and trauma networks can have catchment areas of up to six million people, it is not surprising therefore that current pathways differ from the ‘ideal’ British Society of Rehabilitation Medicine pathway. However, clinical guidelines suggest that all trauma patients should have access to timely and appropriate rehabilitation, including vocational rehabilitation and psychological support.^37^

Not only is there limited vocational rehabilitation service provision across the major trauma networks, there are long waiting lists in areas where there is good support. This means that early and timely rehabilitation after trauma is not always feasible. Evidence supports the benefit of early vocational rehabilitation, which should be delivered as soon as possible post-trauma.^20–25^ The importance of timely psychological support post-trauma is also recognised in the literature^38,39^ and patients should have access to these services to reduce long-term psychological problems. Occupational therapists and other allied health professionals may benefit from training to increase confidence in addressing vocational and psychological issues. However, services and resources need to be available in order that any problems identified can be addressed for the trauma population.

This study had several strengths and provides new evidence to highlight gaps in current service provision post-traumatic injuries. We interviewed a wide range of stakeholders across a variety of trauma networks and obtained a broad perspective of the current pathways across five diverse (i.e. geographically, socioeconomically, and ethnically) trauma networks. To ensure pathway maps were as accurate as possible, they were amended as necessary following stakeholder engagements and their development was an iterative process. We also drew on clinical guidelines to identify missing information and guide questioning when finalising our diagrams against all stakeholder feedback. However, there were also some limitations. We did not manage to recruit any commissioners or employers, and only spoke to a small number of carers, meaning that we were unable to obtain their perspectives on the rehabilitation pathway.

In summary, while there are many examples of rehabilitation identified especially for specific injury groups (e.g. traumatic brain and spinal injuries), our research shows also there are many gaps in service provision, which were more pronounced for other injury groups (e.g. musculoskeletal injuries and amputations) and for patients located further from major trauma centres. The gaps and/or inconsistencies in care were especially problematic in relation to vocational rehabilitation and psychological services across the major trauma networks. Guidelines and literature consistently recommend a multidisciplinary approach to rehabilitation; however, it appears that the system is not as co-ordinated as it could be.

Rehabilitation services in the UK (and likely in other countries) have developed piecemeal, usually in response to specific identified problems such as stroke, spinal cord injury, amputations. This means the system is fragmented and has not developed with a strong theoretical framework, nor has it developed in a patient-centred way. Consequently, there is a complex set of individual services which address specific injury types or problems, but rarely consider the multifaceted rehabilitation needs of a patient. In contrast, patients who have suffered major trauma often present with a broad range of problems which usually require input from multiple individual services. This means that patients frequently have problems for which there is no identified specific service, leading to issues and poor rehabilitation support. A timely example is patients with long-term rehabilitation needs following COVID-19, in which individuals may require pulmonary rehabilitation, but may also need additional input relating to emotional and psychological problems, and possibly fatigue and cognitive issues. Given the complexity of the rehabilitation pathway, it is unlikely a patient will be able to access support for all issues in a timely manner.

Further research is required to map vocational rehabilitation provision and identify service gaps across all major trauma networks in the UK. This should be informed by interviews with a wider range of stakeholders, including carers, employers and commissioners. Research is also required to evaluate the use of the Rehabilitation Prescription and its impact on vocational rehabilitation and psychological support received by trauma patients. Commissioners and providers of rehabilitation services should use our findings to assess how well services are meeting patient need and ensure provision of services addressing the gaps we identified.

**Clinical messages**

- Rehabilitation pathways followed by patients after trauma are extremely complex, with few, if any patients following an ‘ideal’ pathway.
- There is a lack of vocational rehabilitation and psychological support, particularly for individuals with musculoskeletal injuries.
- Continuity of care on discharge from acute to community services is hampered by a lack of communication.

## Supplemental Material

sj-pdf-1-cre-10.1177_0269215520971777 – Supplemental material for A study of mapping usual care and unmet need for vocational rehabilitation and psychological support following major trauma in five health districts in the UK Click here for additional data file. Supplemental material, sj-pdf-1-cre-10.1177_0269215520971777 for A study of mapping usual care and unmet need for vocational rehabilitation and psychological support following major trauma in five health districts in the UK by Jade Kettlewell, Stephen Timmons, Kay Bridger, Denise Kendrick, Blerina Kellezi, Jain Holmes, Priya Patel and Kate Radford in Clinical Rehabilitation

## References

[1] Fisher A Ross C Henderson C, et al. Major trauma care in England, National Audit Office. Statement 2010; 10: 8.

[2] Kendrick D Vinogradova Y Coupland C, et al. Making a successful return to work: the UK burden of injury multicentre longitudinal study. Br J Gen Pract 2012; 62: e82. 10.3399/bjgp12X625139 PMC3268498 22520774

[3] Kendrick D Vinogradova Y Coupland C, et al. Getting back to work after injury: the UK Burden of Injury multicentre longitudinal study. BMC Public Health 2012; 12: 584. 22853715 10.1186/1471-2458-12-584 PMC3444403

[4] Donker-Cools BHPM Daams JG Wind H, et al. Effective return-to-work interventions after acquired brain injury: a systematic review. Brain Injury 2016; 30: 113–131. 26645137 10.3109/02699052.2015.1090014

[5] Roels EH Aertgeerts B Ramaekers D, et al. Hospital-and community-based interventions enhancing (re) employment for people with spinal cord injury: a systematic review. Spinal Cord 2016; 54: 2–7. 26305872 10.1038/sc.2015.133

[6] Saltychev M Eskola M Tenovuo O, et al. Return to work after traumatic brain injury: systematic review. Brain Injury 2013; 27: 1516–1527. 24131314 10.3109/02699052.2013.831131

[7] Karjalainen KA Malmivaara A van Tulder MW, et al. Multidisciplinary biopsychosocial rehabilitation for subacute low-back pain among working age adults. Cochrane Database Syst Rev 2003; 2: CD002193. 10.1002/14651858.CD002193 12804427

[8] Crowther RE Marshall M Bond GR, et al. Helping people with severe mental illness to obtain work: systematic review. BMJ 2001; 322: 204–208. 11159616 10.1136/bmj.322.7280.204 PMC26585

[9] Kendrick D Baker R Hill T, et al. Early risk factors for depression, anxiety and post-traumatic distress after hospital admission for unintentional injury: multicentre cohort study. J Psychosom Res 2018; 112: 15–24. 30097131 10.1016/j.jpsychores.2018.06.008

[10] Hoefsmit N Houkes I Nijhuis FJN. Intervention characteristics that facilitate return to work after sickness absence: a systematic literature review. J Occup Rehab 2012; 22: 462–477. 10.1007/s10926-012-9359-z PMC3484272 22476607

[11] Waddell G Burton AK Kendall NAS. Vocational rehabilitation–what works, for whom, and when?(Report for the vocational rehabilitation task group). London: TSO, 2008.

[12] Hou WH Chi CC Lo HL, et al. Vocational rehabilitation for enhancing return-to-work in workers with traumatic upper limb injuries. Cochrane Database Syst Rev 2017; 12(12): CD010002. 10.1002/14651858.CD010002.pub3 PMC6485969 29210462

[13] British Society of Rehabilitation Medicine. Rehabilitation for patients in the acute care pathway following severe disabling illness or injury: BSRM core standards for specialist rehabilitation. London: British Society of Rehabilitation Medicine, 2014.

[14] British Society of Rehabilitation Medicine. Specialist rehabilitation in the trauma pathway: BSRM core standards. London: BSRM, 2013.

[15] Sinclair E Radford K Grant M, et al. Developing stroke-specific vocational rehabilitation: a soft systems analysis of current service provision. Disabil Rehabil 2014; 36: 409–417. 23692389 10.3109/09638288.2013.793410 PMC3971737

[16] Playford E Radford K Burton C, et al. Mapping vocational rehabilitation services for people with long term neurological conditions: summary report. London: Department of Health, 3 2011.

[17] Hayward K Mateen BA Playford ED, et al. Developing vocational rehabilitation services for people with long-term neurological conditions: identifying facilitators and barriers to service provision. Br J Occup Ther 2019; 82: 337–347.

[18] Scholes J Checkland PB. Soft systems methodology in action. Chichester: Wiley, 1990, pp.876–910.

[19] Braun V Clarke V. Successful qualitative research: a practical guide for beginners. London: Sage, 2013.

[20] Radford K Sutton CJ Sach T, et al. Early, specialist vocational rehabilitation to facilitate return to work after traumatic brain injury: the FRESH feasibility RCT. Health Technol Assess 2018; 22: 1–156. 10.3310/hta22330 PMC6004540 29863459

[21] Radford K Phillips J Drummond A, et al. Return to work after traumatic brain injury: cohort comparison and economic evaluation. Brain Injury 2013; 27: 507–520. 23473058 10.3109/02699052.2013.766929

[22] Fadyl JK McPherson KM. Approaches to vocational rehabilitation after traumatic brain injury: a review of the evidence. J Head Trauma Rehab 2009; 24: 195–212. 10.1097/HTR.0b013e3181a0d458 19461367

[23] Middleton JW Johnston D Murphy G, et al. Early access to vocational rehabilitation for spinal cord injury inpatients. J Rehab Med 2015; 47: 626–631. 10.2340/16501977-1980 26034973

[24] Ntsiea MV Van Aswegen H Lord S, et al. The effect of a workplace intervention programme on return to work after stroke: a randomised controlled trial. Clin Rehab 2015; 29: 663–673. 10.1177/0269215514554241 25322870

[25] Cancelliere C Donovan J Stochkendahl MJ, et al. Factors affecting return to work after injury or illness: best evidence synthesis of systematic reviews. Chiropr Man Ther 2016; 24: 32. 10.1186/s12998-016-0113-z PMC5015229 27610218

[26] Kellezi B Beckett K Earthy S, et al. Understanding and meeting information needs following unintentional injury: comparing the accounts of patients, carers and service providers. Injury 2015; 46: 564–571. 25533126 10.1016/j.injury.2014.11.035

[27] Christie N Beckett K Earthy S, et al. Seeking support after hospitalisation for injury: a nested qualitative study of the role of primary care. Br J Gen Pract 2016; 66: e24–e31. 10.3399/bjgp15X688141 PMC4684032 26639949

[28] Beckett K Earthy S Sleney J, et al. Providing effective trauma care: the potential for service provider views to enhance the quality of care (qualitative study nested within a multicentre longitudinal quantitative study). BMJ Open 2014; 4: e005668. 10.1136/bmjopen-2014-005668 PMC4091464 25005598

[29] Kellezi B Earthy S Sleney J, et al. What can trauma patients’ experiences and perspectives tell us about the perceived quality of trauma care? A qualitative study set within the uk National Health Service. Injury. Epub ahead of print 2020. DOI: 10.1016/j.injury.2020.02.063. 32127201

[30] University of Oslo Institute of Health and Society. Rehabilitation needs, service provision and costs in the first year following traumatic injuries. Institute of Health and Society. https://www.med.uio.no/helsam/english/research/projects/tbi-rehab-first-year/ (2020, accessed 12 October 2020).

[31] Wade DT. What is rehabilitation? An empirical investigation leading to an evidence-based description. London, England: SAGE Publications, 2020. 10.1177/0269215520905112 PMC7350200 32037876

[32] Norlund A Ropponen A Alexanderson K. Multidisciplinary interventions: review of studies of return to work after rehabilitation for low back pain. J Rehab Med 2009; 41: 115–121. 10.2340/16501977-0297 19229442

[33] Trexler LE Parrott DR Malec JF. Replication of a prospective randomized controlled trial of resource facilitation to improve return to work and school after brain injury. Arch Phys Med Rehab 2016; 97: 204–210. 10.1016/j.apmr.2015.09.016 26452718

[34] Abrahamson V Jensen J Springett K, et al. Experiences of patients with traumatic brain injury and their carers during transition from in-patient rehabilitation to the community: a qualitative study. Disabil Rehab 2017; 39: 1683–1694. 10.1080/09638288.2016.1211755 27557977

[35] NCASRI. Final report of the national clinical audit of specialist rehabilitation following major injury. 2019. London: Northwick Park Hospital.

[36] The Trauma Audit & Research Network (TARN). Major trauma rehabilitation prescription 2019 TARN data entry guidance document. NHS England, 2018.

[37] NHS Clinical Advisory Group for Major Trauma. Regional networks for major trauma: NHS clinical advisory groups report. London: Department of Health, 2010.

[38] Regel S Joseph S. Post-traumatic stress. Oxford: Oxford University Press, 2017.

[39] National Institute for Clinical Excellence. Post-traumatic stress disorder: NICE guideline. www.nice.org.uk/guidance/ng116, 2018. 31211536

