# Supplementary material for: A study of mapping usual care and unmet need for vocational rehabilitation and psychological support following major trauma in five health districts in the UK
Source: Clin Rehabil. 2020 Nov 23;35(5):750–64. doi: 10.1177/0269215520971777 (PMC8076839; doi:10.1177/0269215520971777)
Supplement: sj-pdf-1-cre-10.1177_0269215520971777 – Supplemental material for A study of mapping usual care and unmet need for vocational rehabilitation and psychological support following major trauma in five health districts in the UK [file sj-pdf-1-cre-10.1177_0269215520971777.pdf]

Figure 1: The 'ideal' rehabilitation pathway following major traumatic injury taken from the BSRM core standards[14]. *Patients flow through the system from acute care to community care, sometimes requiring more specialist care at a level 1 or 2 inpatient unit.*

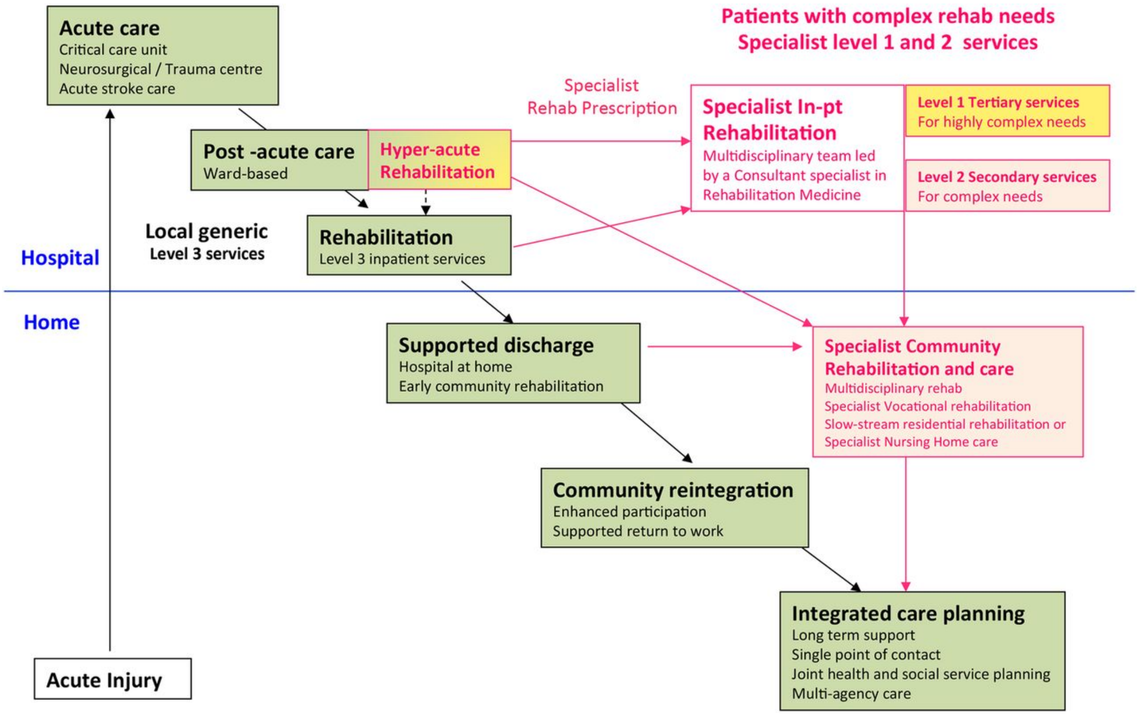

Figure 2: The 'reality' of the trauma pathway; example of rehabilitation pathway, highlighting the current issues. *(ABI: Acquired Brain Injury; GP: General Practitioner; IAPT: Improving Access to Psychological Therapies; MTC: Major Trauma Centre; TBI: Traumatic Brain Injury; PTSD: Post-Traumatic Stress Disorder; VR: Vocational Rehabilitation)* ** We intend for this figure to be presented as an interactive component, so that the issues in the coloured boxes appear when reader hovers over the different rehabilitation services.

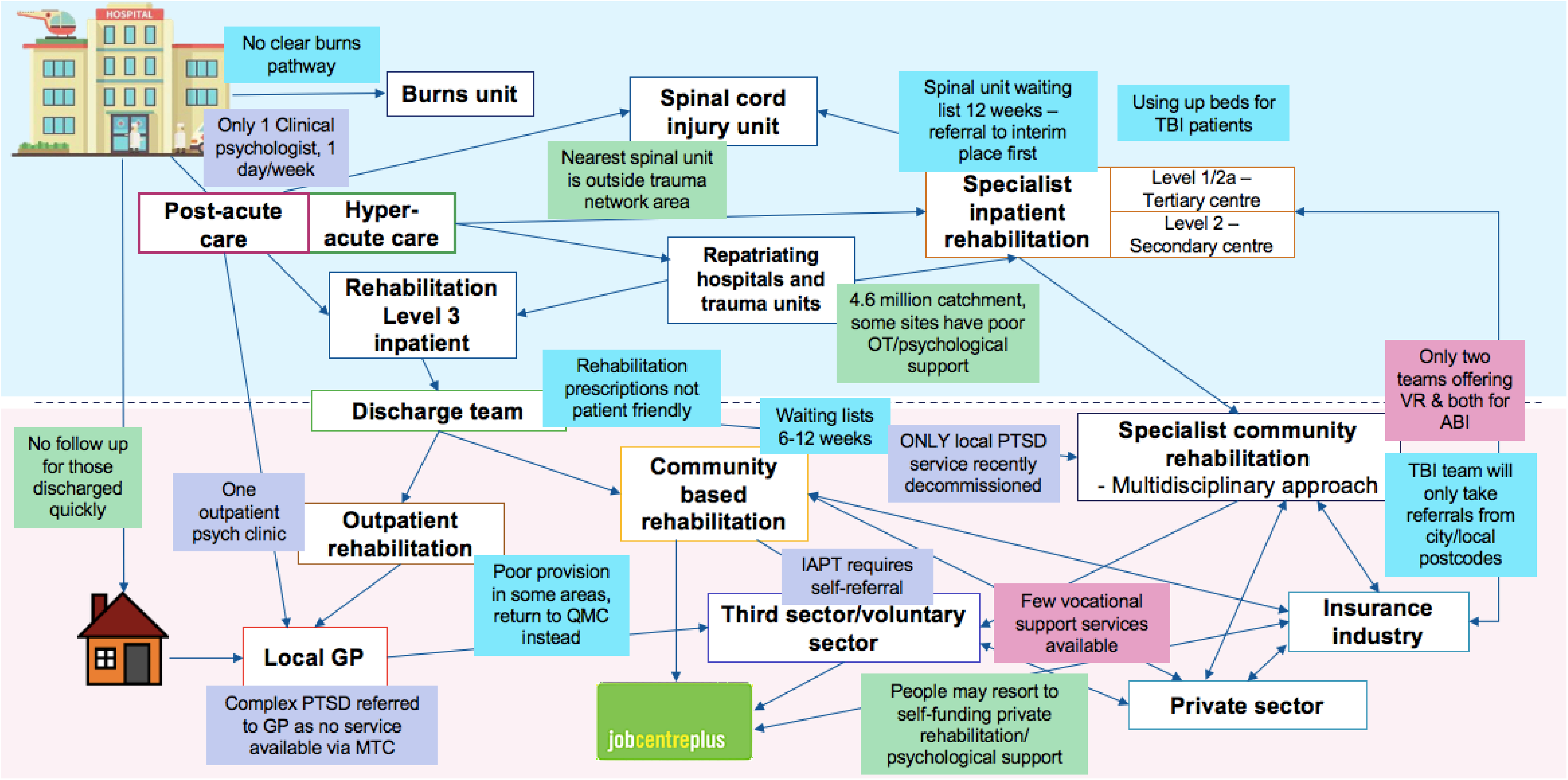

Appendix 1: Summary of resources used

Resource	Reference (link where appropriate)
British Society of Rehabilitation Medicine. Specialist Rehabilitation in the Trauma pathway: BSRM core standards. 2018	British Society of Rehabilitation Medicine. Specialist Rehabilitation in the Trauma pathway: BSRM core standards. November 2018.
British Society of Rehabilitation Medicine. Rehabilitation for patients in the acute care pathway following severe disabling illness or injury: BSRM core standards for specialist rehabilitation. 2014	British Society of Rehabilitation Medicine. Rehabilitation for patients in the acute care pathway following severe disabling illness or injury: BSRM core standards for specialist rehabilitation. 2014. London: British Society of Rehabilitation Medicine
British Society of Rehabilitation Medicine. Specialist Rehabilitation in the Trauma Pathway: BSRM Core Standards. 2013	British Society of Rehabilitation Medicine. Specialist Rehabilitation in the Trauma Pathway: BSRM Core Standards. BSRM London, 2013.
British Society of Rehabilitation Medicine Standards for Rehabilitation Services: Mapped on to the National Service Framework for Long-Term Conditions. 2009	Turner-Stokes L and Ward C. BSRM Standards for Rehabilitation Services: Mapped on to the National Service Framework for Long-Term Conditions. www.bsrm.org.uk: British Society of Rehabilitation Medicine, 2009.
Final report of the National Clinical Audit of Specialist Rehabilitation following major Injury. 2019	NCASRI. Final report of the National Clinical Audit of Specialist Rehabilitation following major Injury. 2019. London: Northwick Park Hospital
First report of the National Clinical Audit of Specialist Rehabilitation following Major Injury. 2016	NCASRI. First report of the National Clinical Audit of Specialist Rehabilitation following Major Injury. 2016. London: Northwick Park Hospital.
Second report of the National Clinical Audit of Specialist Rehabilitation following major Injury. Overcoming the Challenges. 2017	NCASRI. Second report of the National Clinical Audit of Specialist Rehabilitation following major Injury. Overcoming the Challenges. . 2017. London: Northwick Park Hospital.
Neurological Rehabilitation: A Briefing Paper for Commissioners of Clinical Neurosciences. 2008	British Society of Rehabilitation Medicine. Neurological Rehabilitation: A Briefing Paper for Commissioners of Clinical Neurosciences. 2008. British Society of Rehabilitation Medicine.
The Trauma Audit & Research Network (TARN) website.	The Trauma Audit & Research Network (TARN). Performance Comparison: Trauma Care, https://www.tarn.ac.uk/Content.aspx?ca=15 (2019, 2019).
Regional Networks for Major Trauma: NHS Clinical Advisory Groups Report	Regional Networks for Major Trauma, NHS Clinical Advisory Groups Report September 2010 https://www.uhs.nhs.uk/media/suhtinternet/services/emergencymedicine

Appendix 2: Mapping of Rehabilitation Pathways in Trauma Network 1

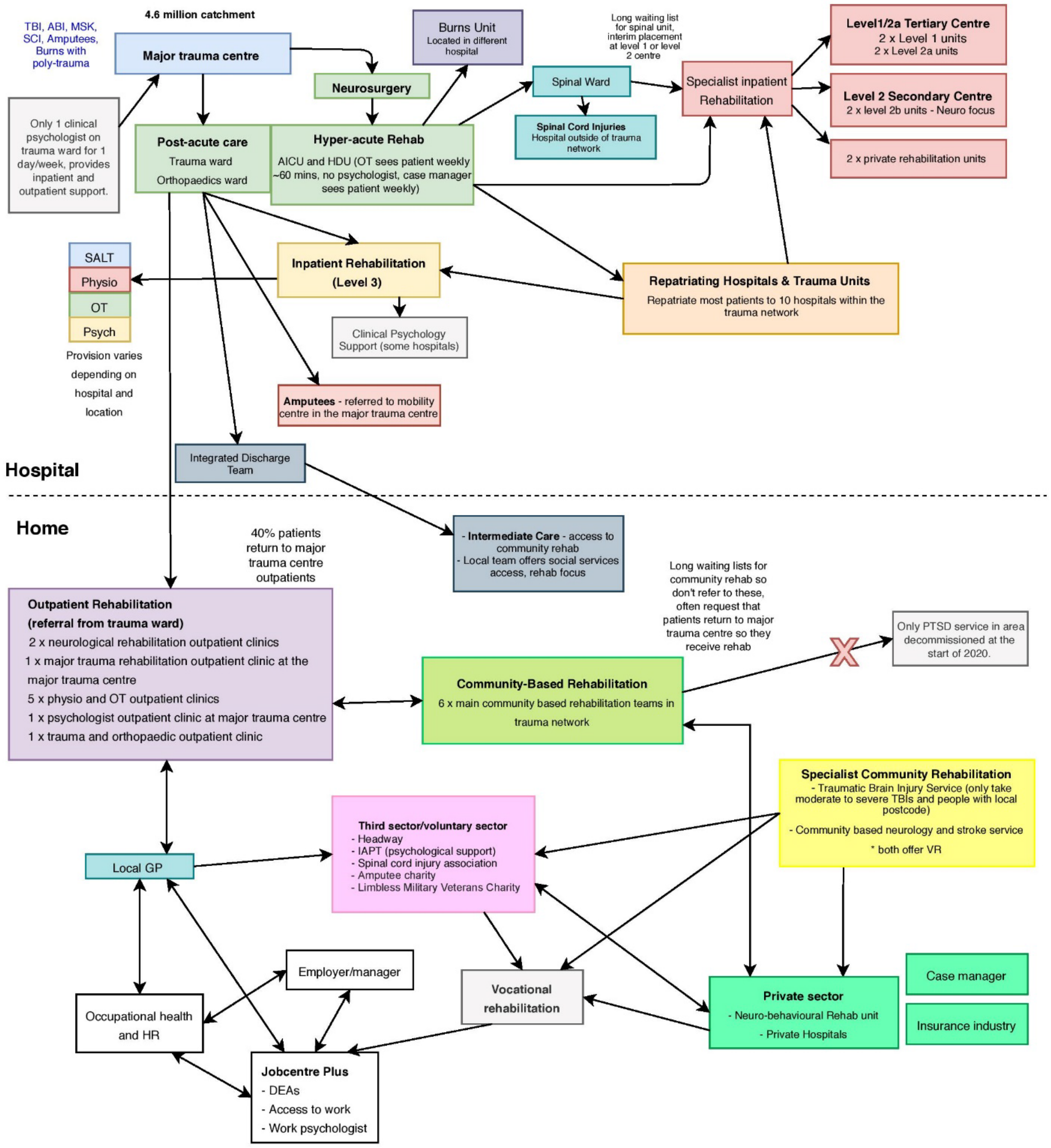

ABI: Acquired Brain Injury; AICU: Adult Intensive Care Unit; DEA: Disability Employment Advisor; GP: General Practitioner; HDU: High Dependency Unit; HR: Human Resources; IAPT: Improving Access to Psychological Therapies; OT: Occupational Therapy; MSK: Musculoskeletal; Physio: physiotherapy; Psych: Clinical psychologist/psychological support; PTSD: Post-Traumatic Stress Disorder; Rehab: Rehabilitation; SALT: Speech and Language Therapy; SCI: Spinal Cord Injury; TBI: Traumatic Brain Injury; VR: Vocational Rehabilitation

Appendix 3: Mapping of Rehabilitation Pathways in Trauma Network 2

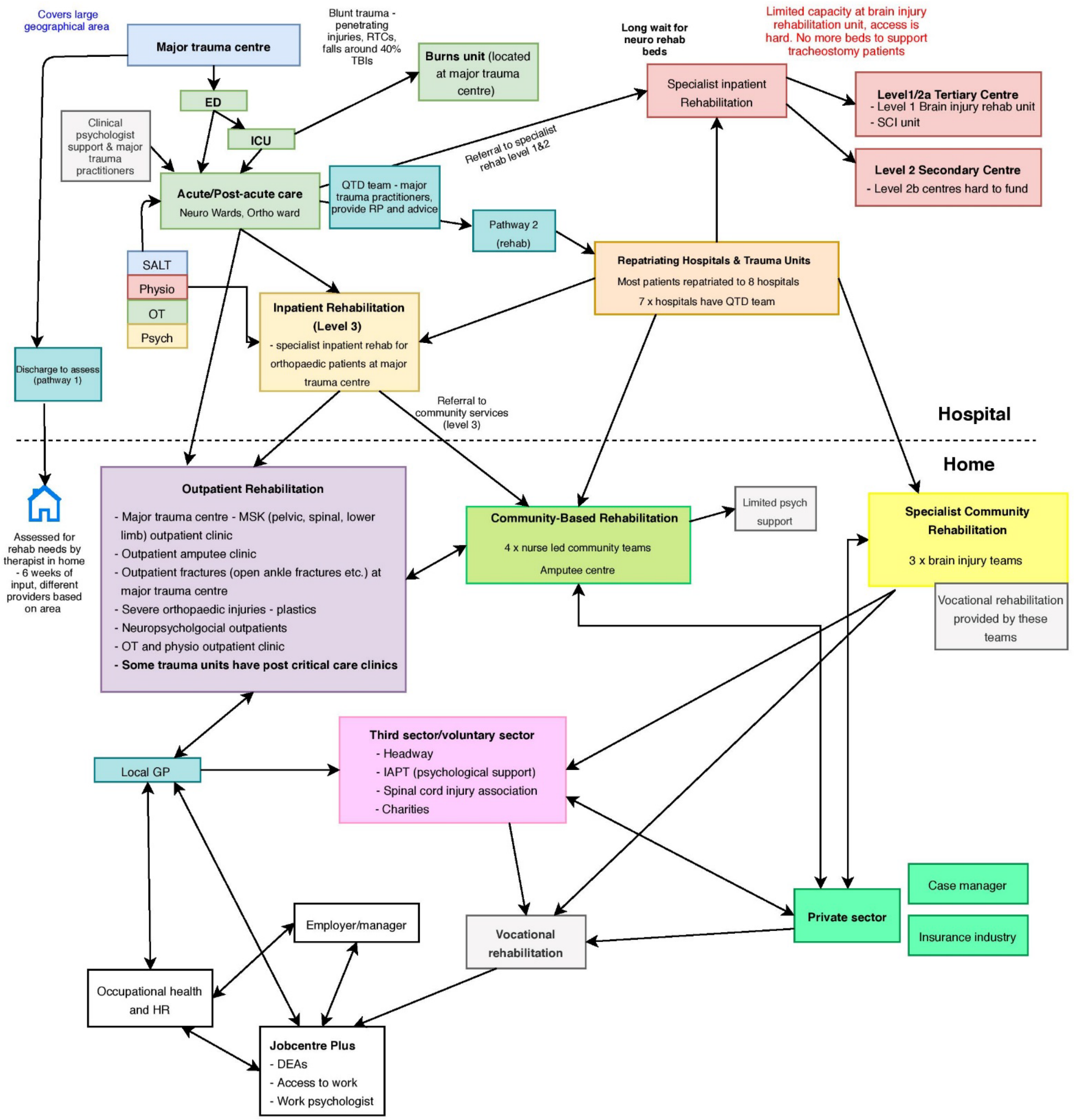

Disability Employment Advisor; ED: Emergency Department; GP: General Practitioner; HR: Human Resources; IAPT: Improving Access to Psychological Therapies; ICU: Intensive Care Unit; Ortho: Orthopaedic; OT: Occupational Therapy; Physio: physiotherapy; Psych: Clinical psychologist/psychological support; Rehab: Rehabilitation; RP: Rehabilitation Prescription; RTC: Road Traffic Collision; QTD: Quality Trauma Discharge; SALT: Speech and Language Therapy; SCI: Spinal Cord Injury; TBI: Traumatic Brain Injury

Appendix 4: Mapping of Rehabilitation Pathways in Trauma Network 3

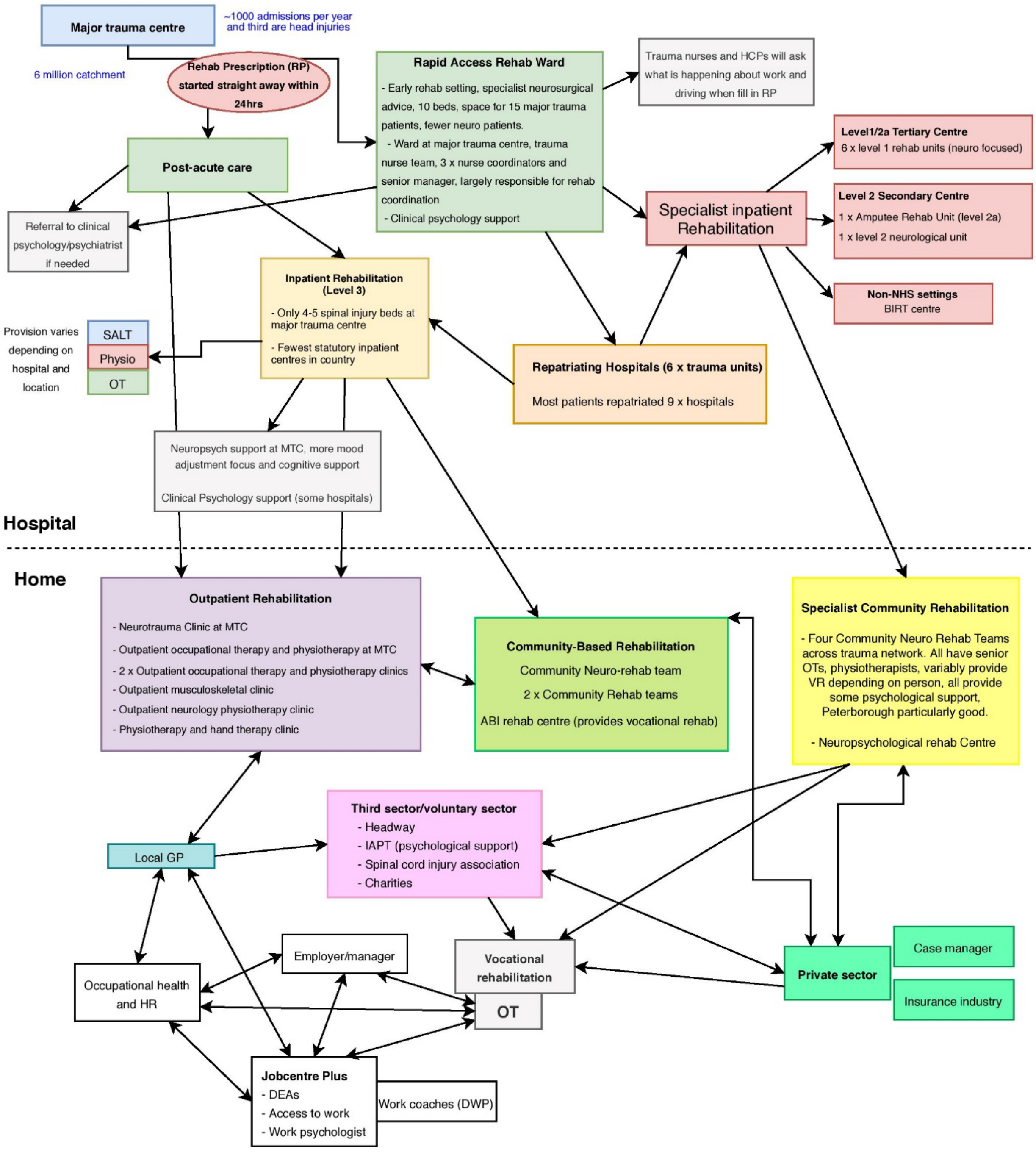

DEA: Disability Employment Advisor; DWP: Department for Work and Pensions; GP: General Practitioner; HCPs: Healthcare Professionals; HR: Human Resources; IAPT: Improving Access to Psychological Therapies; MTC: Major Trauma Centre; OT: Occupational Therapy; Physio: physiotherapy; Rehab: Rehabilitation; RP: Rehabilitation Prescription SALT: Speech and Language Therapy; VR: Vocational Rehabilitation

Appendix 5: Mapping of Rehabilitation Pathways in the Trauma Network 4

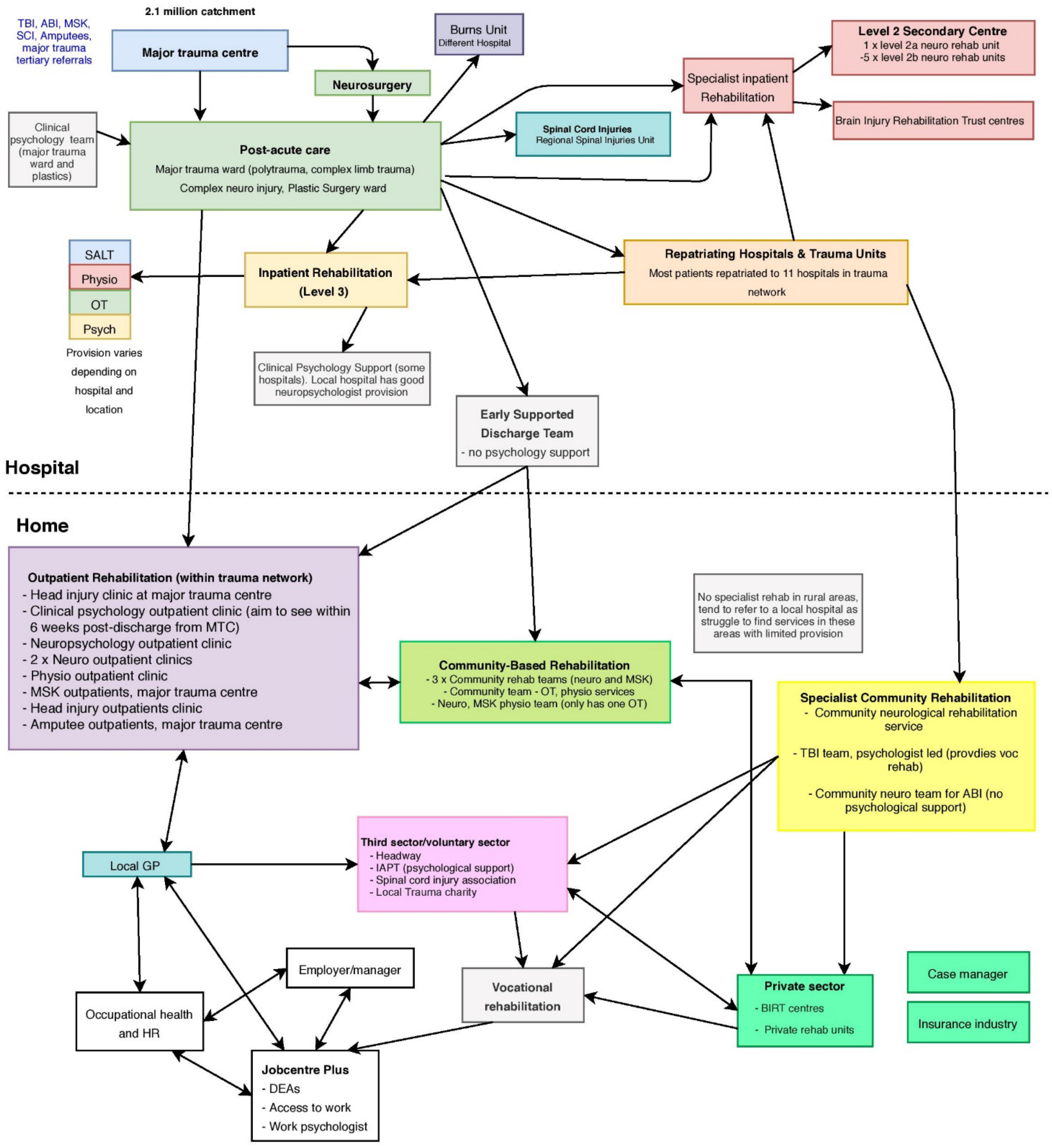

ABI: Acquired Brain Injury; DEA: Disability Employment Advisor; GP: General Practitioner; HR: Human Resources; IAPT: Improving Access to Psychological Therapies; OT: Occupational Therapy; MSK: Musculoskeletal; MTC: Major Trauma Centre; Physio: physiotherapy; Psych: Clinical psychologist/psychological support; PTSD: Post-Traumatic Stress Disorder; Rehab: Rehabilitation; SALT: Speech and Language Therapy; SCI: Spinal Cord Injury; TBI: Traumatic Brain Injury

Appendix 6: Mapping of Rehabilitation Pathways in Trauma Network 5

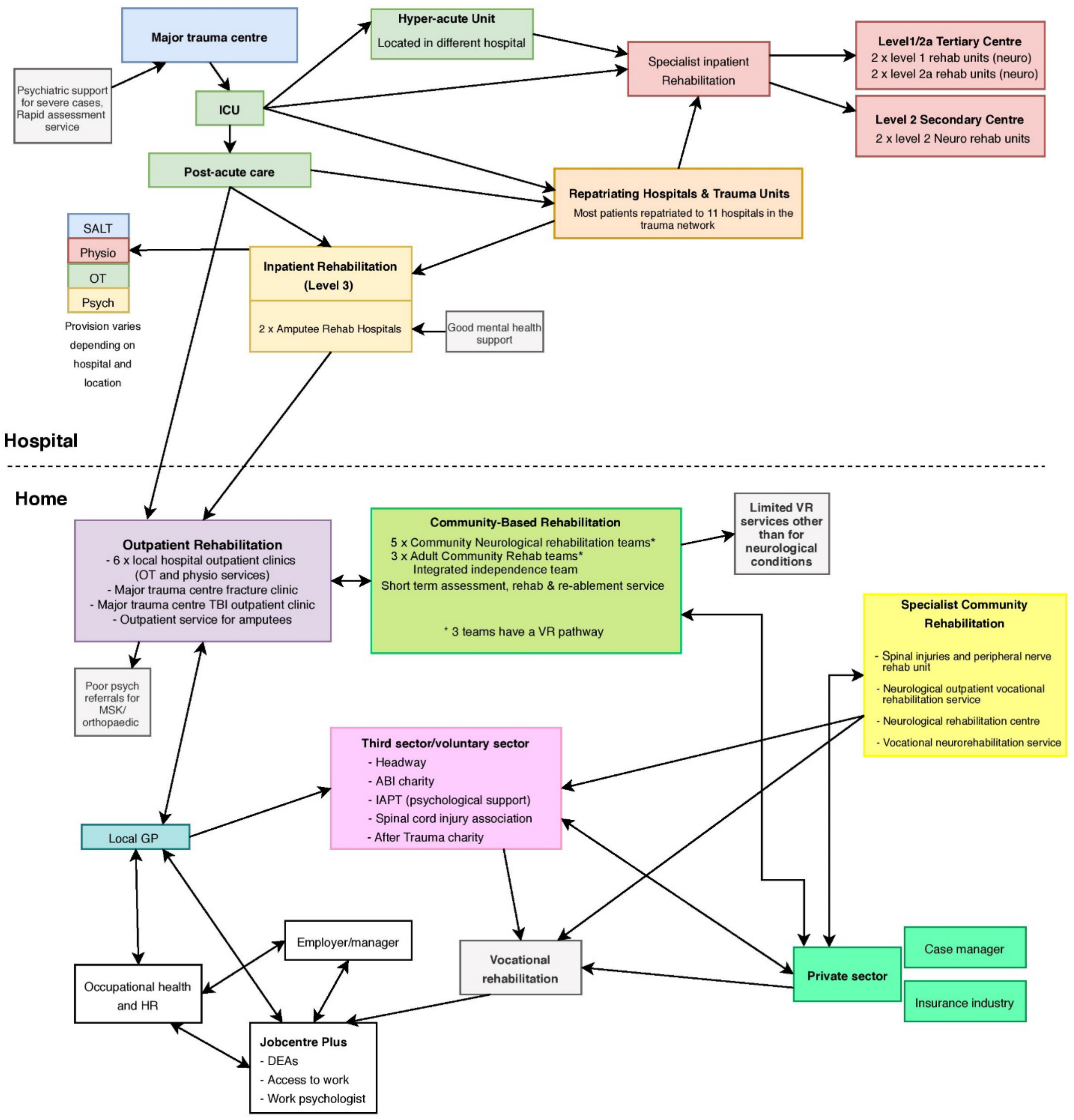

ABI: Acquired Brain Injury; DEA: Disability Employment Advisor; GP: General Practitioner; HR: Human Resources; IAPT: Improving Access to Psychological Therapies; ICU: Intensive Care Unit; OT: Occupational Therapy; Physio: physiotherapy; Psych: Clinical psychologist/psychological support; Rehab: Rehabilitation; SALT: Speech and Language Therapy; SCI: Spinal Cord Injury; TBI: Traumatic Brain Injury; VR: Vocational Rehabilitation
